# An *in vitro* visual study of fugitive aerosols released during aerosol therapy to an invasively ventilated simulated patient

**DOI:** 10.1080/10717544.2021.1951893

**Published:** 2021-07-14

**Authors:** Marc Mac Giolla Eain, Mary Joyce, Ronan MacLoughlin

**Affiliations:** a Research and Development, Science and Emerging Technologies, Aerogen, Galway, Ireland; b School of Pharmacy and Biomolecular Sciences, Royal College of Surgeons, Dublin, Ireland; c School of Pharmacy and Pharmaceutical Sciences, Trinity College, Dublin, Ireland

**Keywords:** COVID-19, pressurized metered dose inhaler, jet nebulizer, vibrating mesh nebulizer, fugitive emissions, invasive ventilation, aerosol therapy, Schlieren imaging

## Abstract

COVID-19 can cause serious respiratory complications resulting in the need for invasive ventilatory support and concurrent aerosol therapy. Aerosol therapy is considered a high risk procedure for the transmission of patient derived infectious aerosol droplets. Critical-care workers are considered to be at a high risk of inhaling such infectious droplets. The objective of this work was to use noninvasive optical methods to visualize the potential release of aerosol droplets during aerosol therapy in a model of an invasively ventilated adult patient. The noninvasive Schlieren imaging technique was used to visualize the movement of air and aerosol. Three different aerosol delivery devices: (i) a pressurized metered dose inhaler (pMDI), (ii) a compressed air driven jet nebulizer (JN), and (iii) a vibrating mesh nebulizer (VMN), were used to deliver an aerosolized therapeutic at two different positions: (i) on the inspiratory limb at the wye and (ii) on the patient side of the wye, between the wye and endotracheal tube, to a simulated intubated adult patient. Irrespective of position, there was a significant release of air and aerosol from the ventilator circuit during aerosol delivery with the pMDI and the compressed air driven JN. There was no such release when aerosol therapy was delivered with a closed-circuit VMN. Selection of aerosol delivery device is a major determining factor in the release of infectious patient derived bioaerosol from an invasively mechanically ventilated patient receiving aerosol therapy.

## Introduction

COVID-19 is a highly contagious respiratory disease caused by the novel severe acute respiratory syndrome coronavirus 2 (SARS-CoV-2) virus. The severity of the disease and required treatment, ranges from asymptomatic, requiring no treatment, to life threatening, requiring intensive care and respiratory support (Yang et al., 2020). The primary care strategy for COVID-19 patients is respiratory support, with the use of high flow oxygen therapy (HFOT) one of the preferred options (Li et al., 2020). Conversely, several studies have shown that invasive mechanical ventilation still remains high among COVID-19 patients (Ferrando et al., 2020; Grasselli et al., 2020). Aerosol therapy is the most effective means of delivering lung targeted therapeutics to patients receiving respiratory support. Aerosolized therapeutics can be delivered with pressurized metered dose inhalers (pMDIs), dry powder inhalers, and nebulizers, with compressed air driven jet nebulizers (JNs) and vibrating mesh nebulizers (VMNs) the most common nebulizers used in the critical care environment (Ehrmann et al., 2013). However, aerosol therapy is considered a risk for viral transmission and cross infection between patients and healthcare professionals (Razzak et al., 2020).

To mitigate against the potential viral transmission and cross infection between patients and healthcare professionals, a number of clinical guidance documents and papers have been published within the last year (Alhazzani et al., 2020; Ari, 2020; Fink et al., 2020; Respiratory Care Committee of Chinese Thoracic Society, 2020; Cazzola et al., 2021). However, the recommendations in these guidance documents are often contradictory with one another, GINA recommends against the use of nebulizers (Global Initiative for Asthma, 2020) while the International Society for Aerosols in Medicine (Fink et al., 2020), the British Thoracic Society (British Thoracic Society, 2021) support the use of nebulizers, provided the respiratory circuit is not broken or opened.

The majority of studies in this area of aerosol therapy to COVID-19 patients have focused on the potential release and dispersion of bioaerosol from potentially infected patients receiving HFOT (McGrath et al., 2019; Li et al., 2020). However, little is known about the potential release of patient derived bioaerosol from invasively ventilated patients. This work aims to address this knowledge gap. Using the well-established Schlieren imaging technique, a visual study was completed to determine if and where potential bioaerosol emission occurs during the delivery of an aerosolized therapeutic to a simulated intubated mechanically ventilated model adult patient.

## Materials and methods

### Respiratory circuit

**Figure 1.**
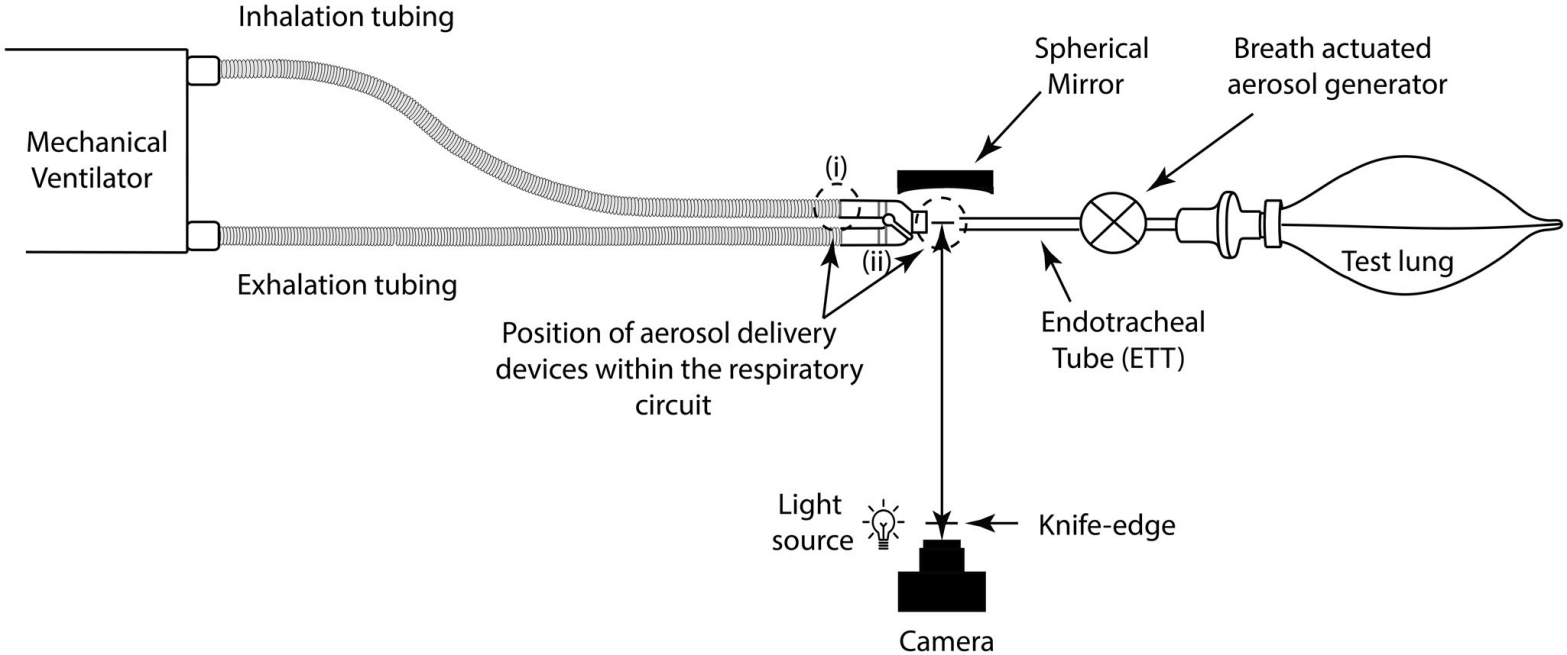
Schematic illustration of the experimental setup used in this study.

Figure 1 presents a schematic illustration of the experimental facility used in this study. It consists of a critical care mechanical ventilator (Bellavista, IMT Medical, Buchs, Switzerland) with a dual limb respiratory circuit (RT380 Fisher & Paykel, Auckland, New Zealand) which simulated a healthy adult ventilation pattern (BR = 15 BPM, Vt = 500 mL, I:E: 1.0:1.0). Aerosol delivery was examined at two commonly used positions in the respiratory circuit, (i) on the inspiratory limb at the wye and (ii) at the patient side of the wye, between the wye and an endotracheal tube (ETT) (8.0 mm, Flexicare, Maisemore, UK). A breath actuated aerosol generator was positioned between the ETT and the artificial test lung (IMT Medical, Buchs, Switzerland). The breath actuated aerosol generator was used to generate simulated exhaled patient bioaerosol using a simple saline solution (0.9%, Braun, Melsungen, Germany), on the peak expiratory flow of the breath.

### Aerosol therapy devices

Experiments were performed using a pMDI (Ventolin Evohaler 100 µg pressurized inhaler, GSK, Dublin, Ireland), a compressed air driven JN (Cirrus 2, Intersurgical, Wokingham, UK, at 8 LPM) and a VMN (Aerogen Solo, Aerogen Ltd, Galway, Ireland). An adapter was used to connect the pMDI to the respiratory circuit, the MiniSpacer Dual Spray MDI Adapter (Teleflex Medical, Wayne, PA), at position (i) and the integrated MDI port in the respiratory circuit wye (RT380 Fisher & Paykel, Auckland, New Zealand) was used at position (ii). The JN and VMN were connected to the respiratory circuit by means of a T-piece adaptor (22M/15F Intersurgical, Intersurgical, Wokingham, UK, for the JN and the Aerogen T-piece for the VMN).

### Schlieren optical system

The Schlieren optical system in this study used a 200 mm diameter spherical mirror with a 1 m focal length. An LED with a variable aperture was used as a light source to illuminate the spherical mirror and the image of the mirror was focused onto a knife edge. The light source and knife-edge were placed at the center of curvature of the spherical mirror, which was twice the focal length. 800 × 600-pixel videos were recorded using a monochromatic camera (Phantom v310, Vision Research, Wayne, NJ) with a Nikon Micro-Nikkor f105mm lens. Video files were recorded at a frame rate of 400 frames/second at an exposure of 1 ms. *Post hoc* processing was completed using the Phantom Cineviewer Software (Version 3.5, Vision Research, Wayne, NJ). In this study, the Schlieren optical system revealed the density gradients in the air and integrated this information in the direction of the optical axis in order to produce a planar image of the respective flow patterns. This flow visualization technique was chosen as it is well established in science and engineering, has been used in infection control research since the late 1960s (Lewis et al., 1969; Clark & Edholm, 1985; Clark & De Calcina-Goff, 2009), and allows 3D flow information to be integrated onto a single plane. Furthermore, the Schlieren optical method does not require the use of any tracer gasses or particles, or high-intensity lasers.

## Results and discussion

**Figure 2.**
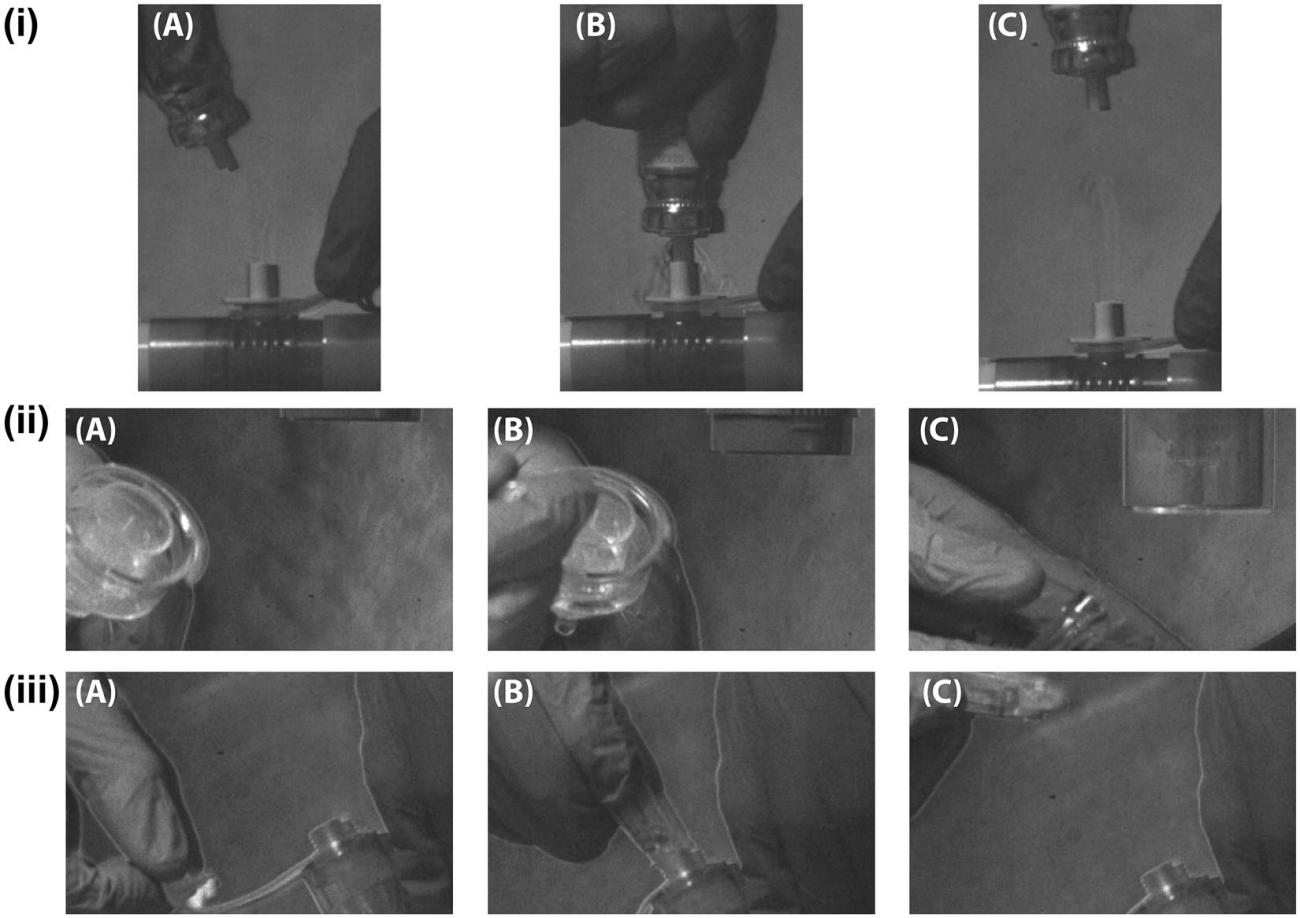
Magnified high-speed images of aerosol therapy delivered with a (i) pressurized metered dose inhaler, (ii) compressed air driven jet nebulizer, and (iii) vibrating mesh nebulizer to a simulated, intubated, mechanically ventilated adult patient. The frames focus on the devices at the time periods immediately (A) pre, (B) during, and (C) post aerosol therapy. The aerosol therapy devices were positioned on the inspiratory limb at the wye of the dual limb respiratory circuit.

Figure 2 presents a series of the high-speed magnified Schlieren video images of the delivery of concurrent aerosol therapy to a simulated intubated adult patient via a (i) pMDI, (ii) JN, and (iii) VMN. These magnified images highlight the main findings of this visual study. A series of images summarizing the overall therapeutic aerosol delivery process, consistent with clinical practice, are presented in supplementary Figures 1 and 2. The therapeutic was delivered on the inspiratory limb at the wye of the dual limb respiratory circuit. The frames, (A–C), within each subfigure, (i–iii), were captured immediately prior to, during and post-delivery of the therapeutic. It is evident from the subfigures and frames presented in Figure 2, that the respiratory circuit must be opened to the environment to deliver aerosol therapy with the pMDI and JN, and there is air released from the respiratory circuit into the environment, Figure 2(i) and (ii) (A–C) Unlike in cases (i) and (ii), there is no release of air from the circuit when the VMN was used to deliver aerosol therapy, Figure 2(iii) (A–C).

**Figure 3.**
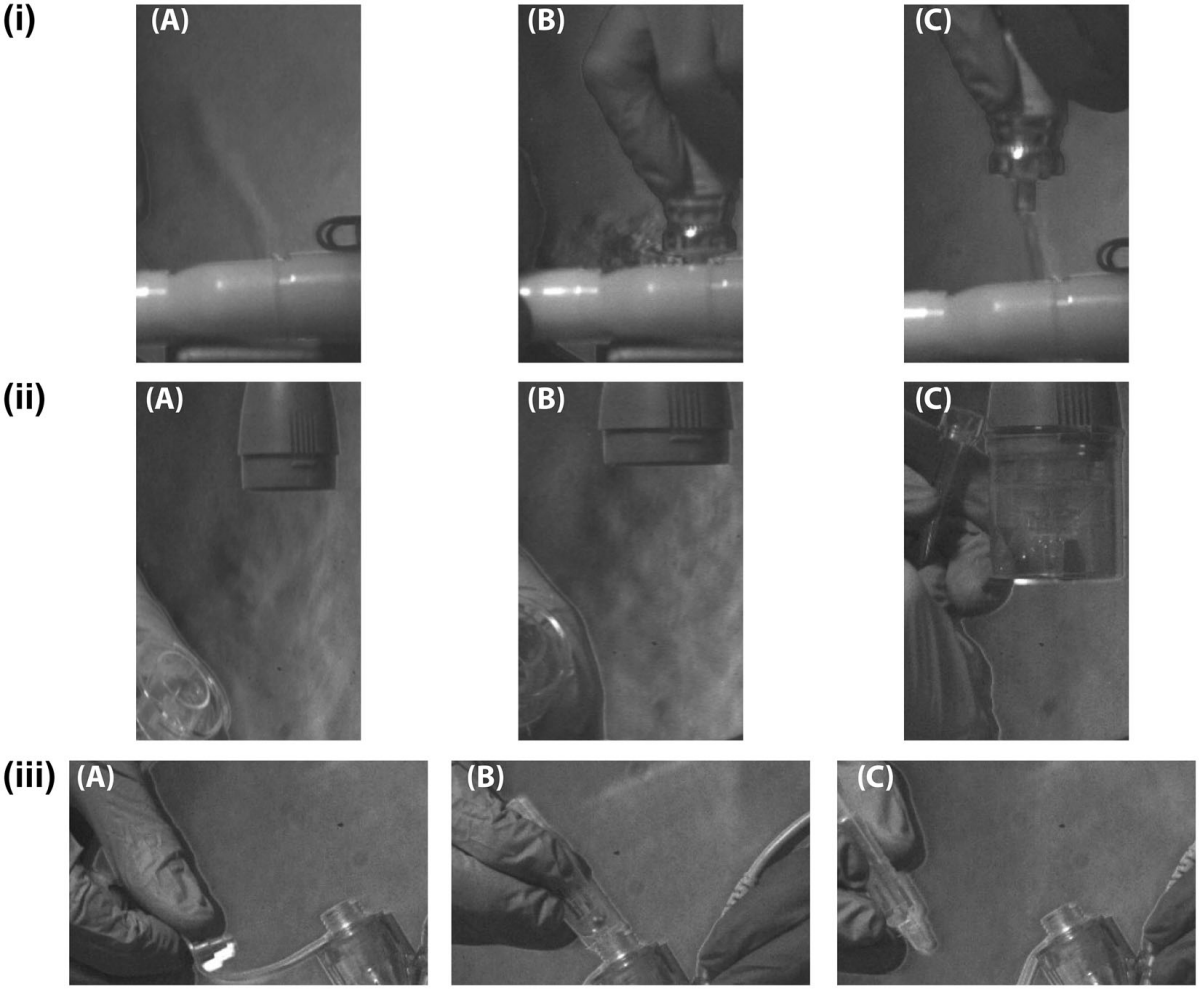
Magnified high-speed images of aerosol therapy delivered with a (i) pressurized metered dose inhaler, (ii) compressed air driven jet nebulizer, and (iii) vibrating mesh nebulizer to a simulated, intubated, mechanically ventilated adult patient. The frames focus on the devices at the time periods immediately (A) pre, (B) during, and (C) post aerosol therapy. The aerosol therapy devices were positioned on the patient side of the wye, between the wye and ETT of the dual limb respiratory circuit.

Figure 3 shows the same stages of therapeutic delivery via a (i) pMDI, (ii) JN, and (iii) VMN; however, the delivery position is on the patient side of the wye junction, between the wye and the ETT. Similar to Figure 2, there is air released into the environment from respiratory circuit during aerosol delivery with the pMDI, Figure 3(i) (A–C) and the JN, Figure 3(ii) (A–C), and none from the VMN, Figure 3(iii) (A–C).

Both Figures 2 and 3 show a release of air from the circuit when opened to deliver the therapeutic with the pMDI and JN. Given the composition of the experimental setup used in this study, the incorporation of the breath actuated aerosol generator, it stands to reason that the air escaping the respiratory circuit is a combination of fugitive medical aerosol, air from the ventilator and simulated patient derived bioaerosol. There are several different devices through which the pMDI and JN can be connected to the respiratory circuit and deliver an aerosolized therapeutic. These include valved holding chambers, reservoirs, valved, and various configurations of in-line adaptors for the pMDI and various T-piece adaptors for the JN. Works by Bishop et al. (1990), Rau et al. (1992), and Diot et al. (1995) among others have compared the aerosol dose delivery efficiency of a number of these different actuators and connectors. However, irrespective of the device used to actuate or connect the pMDI or JN to the respiratory circuit, it is necessary to break or open the respiratory circuit to the environment to deliver the therapeutic agent, which allows the release of these fugitive medical aerosols, ventilator air and patient derived bioaerosol into the environment. Furthermore, by breaking the respiratory circuit as is necessary when delivering aerosol therapy with the pMDI and JN, there is also the possibility that the healthcare worker could contaminate the device and infect the patient with a potential pathogen (Dhand & Li, 2020). By design, this is not possible with the VMN as the medication cup is separate from the respiratory circuit.

Maintaining a closed pressurized circuit during mechanical ventilation is critical in ensuring the safe ventilation of a patient but also in preventing the release of fugitive medical and patient derived bioaerosol. Joyce et al. (2021) used an aerosol particle sizer (APS) to measure the patient derived bioaerosol released from a dual limb mechanically ventilated circuit during nebulizer refill with a VMN and JN. The authors found that there was a significant release of patient derived bioaerosol during refill of the JN, median above ambient levels 710 particles per cm^3^, while levels measured during refill of the VMN were similar to those measured during ambient conditions, median levels above ambient 0 particles per cm^3^. These quantitative measurements affirm the qualitative measurements presented in this piece of work. Of the few remaining studies that have examined fugitive emissions from invasive mechanically ventilated patients (Ari et al., 2016; O’Toole et al., 2020), these studies focused specifically on fugitive medical aerosol released into the environment during an aerosol treatment, no potential patient element was incorporated. To the best of our knowledge, this is the first study to visualize the release of aerosol, both patient derived and medical, into the environment during an aerosol treatment in a clinically representative, simulation of an intubated mechanically ventilated patient.

It should be noted there are a number of limitations to this study. These include: only a single patient model and breath type were considered in this study, only a single type of pMDI and JN were considered, and only one pMDI actuator/adaptor was used. As stated previously there are several other actuator/adaptors that can be used and should be considered in future works. The imaging technique used does not provide any quantitative data or diagnose whether the air released into the environment is patient derived bioaerosol, fugitive medical aerosol, or air from the ventilator.

## Conclusions

This original study provides valuable information to caregivers and policy makers on the best practice for the delivery of aerosolized therapeutics to mechanically ventilated patients with potentially infections respiratory diseases, such as COVID-19 and goes toward supporting the published guidelines that call for use of a closed-circuit nebulizer during aerosol therapy. This study visually demonstrated the release of potentially infected patient derived bioaerosol, fugitive medical aerosol, and ventilator air from the respiratory circuit during the delivery of an aerosolized therapeutic with a pMDI and JN. No such release occurred using a VMN. This study demonstrates the importance of medical aerosol delivery device in mitigating the risk of potentially infectious patient derived bioaerosol release into the environment during aerosol therapy to mechanically ventilated patients. Furthermore, this work supports the conclusions of the consensus guidelines that recommend the use of closed-circuit nebulizers, such as VMNs, and against the use of open circuit devices, such as pMDIs and air compressor driven JNs.
